# Protocol for the CABG-PRIME Study (Coronary Artery Bypass Graft—Platelet Response and Improvement in Medicine Efficacy)—An Exploratory Study to Review the Role of Platelet Function Testing in Improving Patient Outcomes Post-CABG Surgery

**DOI:** 10.3390/jcdd13010035

**Published:** 2026-01-08

**Authors:** Maria Comanici, Anonna Das, Charlene Camangon, Kavya Kanchirassery, Harsimran Singh, Nicholas James Lees, Diana Gorog, Nandor Marczin, Shahzad G. Raja

**Affiliations:** 1 Department of Cardiac Surgery, Harefield Hospital, Royal Brompton and Harefield Hospitals, Part of Guy’s and St Thomas NHS Trust, London UB9 6JH, UK; 2 Department of Anaesthesia and Critical Care, Harefield Hospital, Royal Brompton and Harefield Hospitals, Part of Guy’s and St Thomas NHS Trust, London UB9 6JH, UK; 3 Department of Cardiology, Royal Brompton and Harefield Hospitals, Part of Guy’s and St Thomas NHS Trust, London SW3 6NP, UK; 4 Department of Surgery and Cancer, Faculty of Medicine, Imperial College London, London SW7 2AZ, UK; 5 Division of Anaesthesia, Pain Medicine and Intensive Care, Department of Surgery and Cancer, Imperial College London, London SW7 2BX, UK; 6 Department of Anaesthesia and Intensive Care, Semmelweis University, 1082 Budapest, Hungary

**Keywords:** coronary artery bypass grafting, antiplatelets, personalised medicine

## Abstract

Background: Coronary artery bypass grafting (CABG) is a well-established revascularization strategy for patients with multivessel coronary artery disease. The effectiveness of CABG is significantly influenced by antiplatelet therapy aimed at maintaining graft patency and reducing thrombotic complications. However, substantial inter-individual variability exists in platelet function responses to standard therapies such as aspirin and clopidogrel, leading to antiplatelet resistance. This variability has been linked to increased risks of myocardial infarction, stroke, and early graft failure. Platelet function testing (PFT) offers a potential strategy to identify resistance and guide more personalized antiplatelet therapy. This study aims to evaluate the association between perioperative platelet function test results and clinical outcomes following CABG. By assessing platelet responsiveness at multiple timepoints and correlating findings with postoperative events, the study seeks to determine whether PFT can stratify risk and improve patient management. Methods: This is a prospective, single-centre, observational cohort study conducted at a tertiary NHS cardiac surgery centre. Patients having elective or urgent isolated CABG will be enrolled and undergo perioperative PFT using the TEG6s system. Clinical outcomes will be monitored for 12 months postoperatively, with primary endpoints assessing the correlation between platelet function results and major adverse cardiovascular and cerebrovascular events (MACCE). Secondary endpoints will include the prevalence of antiplatelet resistance, demographic predictors, and the feasibility of integrating PFT into clinical workflows. Results: This study will report the prevalence of aspirin and clopidogrel resistance in CABG patients based on TEG6s PFT, as well as the correlation between platelet function results and MACCE, postoperative bleeding, and the need for surgical re-exploration. Additionally, it will examine the associations between demographic and clinical factors—such as diabetes status, renal function, BMI, and surgical technique—and variability in platelet responsiveness. The feasibility of incorporating PFT into perioperative workflows will also be evaluated, assessing whether results could support personalized antiplatelet management in future clinical trials. Conclusions: Findings from this study will provide real-world evidence regarding platelet function variability in CABG patients and suggest that PFT may identify those at increased risk of thrombotic complications. This exploratory analysis supports the need for larger interventional trials aimed at optimizing individualized postoperative antiplatelet therapy to improve surgical outcomes.

## 1. Introduction

Coronary artery disease remains one of the leading causes of morbidity and mortality worldwide, despite advancements in medical and interventional therapies [1]. Coronary artery bypass grafting (CABG) is a well-established surgical approach for patients with multivessel or complex coronary artery disease, offering durable revascularization and improved long-term outcomes compared to percutaneous coronary intervention in select patient populations [2]. However, the success of CABG is contingent on maintaining graft patency and minimizing postoperative thrombotic complications, which are heavily influenced by perioperative antiplatelet therapy [3].

Aspirin and clopidogrel, the most commonly prescribed antiplatelet agents in coronary artery bypass grafting patients, exert their effects by irreversibly inhibiting platelet cyclooxygenase-1 and P2Y12 receptors, respectively. Despite their widespread use, there is mounting evidence of substantial inter-individual variability in response to these agents, a phenomenon referred to as antiplatelet resistance [4]. Meta-analytic data suggest that aspirin resistance may affect up to 38 percent of patients, while clopidogrel resistance has been observed in up to 40 percent of coronary artery bypass grafting recipients [5]. This variability in platelet responsiveness has been linked to an increased risk of adverse cardiovascular events, including myocardial infarction, stroke, and early graft failure [6].

Platelet function testing (PFT) offers a potential strategy for identifying patients with antiplatelet resistance and guiding personalized therapy to mitigate thrombotic risk. Technologies such as light transmission aggregometry, VerifyNow, impedance aggregometry, and thromboelastography allow for real-time assessment of platelet reactivity, thereby providing objective measures of antiplatelet efficacy [7]. However, despite the technological feasibility, routine use of PFT in CABG pathways remains limited due to the lack of prospective, outcome-based evidence supporting its clinical utility.

Current international guidelines do not offer specific recommendations on personalized antiplatelet strategies post-coronary artery bypass grafting, in part due to this evidence gap [2]. A recent UK-wide survey of cardiac surgeons found significant variability in antiplatelet prescribing practices, reinforcing the need for robust data to inform clinical decision-making in surgical patients [8].

This observational study seeks to address the unmet need for robust data linking perioperative platelet function test (PFT) results with clinical outcomes following CABG. The primary objective of this study is to determine the association between perioperative platelet function test results and clinical outcomes, including major adverse cardiovascular and cerebrovascular events (MACCE), postoperative bleeding, and the need for surgical re-exploration. In this study, MACCE is defined as a composite of myocardial infarction, non-fatal stroke, and cardiovascular death, assessed at 30 days and one year following CABG. The secondary objectives include assessing the prevalence of aspirin and clopidogrel resistance in CABG patients using platelet mapping, identifying demographic and clinical predictors of antiplatelet resistance—such as age, sex, diabetes, renal function, ethnicity, BMI, and surgical technique—and evaluating temporal trends in platelet responsiveness from the preoperative baseline through early and late postoperative periods. Additionally, the study will explore the relationship between high platelet reactivity and the need for postoperative escalation of care, including ICU readmission, inotropic support, and transfusions. Lastly, it will assess the feasibility of integrating PFT into the perioperative care pathway to support individualized antiplatelet management in future interventional studies.

## 2. Methods

### 2.1. Study Design

This is a prospective, single-centre, observational cohort study designed to evaluate the association between perioperative platelet function and clinical outcomes in patients undergoing elective and urgent CABG. The primary objective is to assess variability in platelet responsiveness to standard antiplatelet therapy and its correlation with MACCE, postoperative bleeding, and the need for surgical re-exploration.

#### 2.1.1. Overall Design

The study will be conducted at a single tertiary NHS cardiac surgery centre, enrolling 100 patients who will receive standard perioperative and postoperative care according to institutional and national guidelines. No study drug or investigational device will be tested. The study aims to generate real-world data that may inform future interventional trials or guideline updates regarding the role of PFT in optimizing antiplatelet therapy for surgical patients.

A formal sample size calculation was performed to ensure sufficient statistical power to detect meaningful variability in platelet function and its association with clinical outcomes. Assuming a moderate effect size (Cohen’s d = 0.5), a two-sided alpha level of 0.05, and 80% statistical power, the minimum required sample size for comparison of platelet function measures was estimated at 63 patients.

To account for an anticipated attrition rate of approximately 20%, due to loss to follow-up or incomplete PFT across multiple perioperative timepoints, the adjusted minimum sample size was calculated to be approximately 80 patients. To further enhance robustness and allow for exploratory subgroup and longitudinal analyses, a final target sample size of 100 patients was selected.

#### 2.1.2. Study Design Summary

- Design type: Prospective, observational, non-randomised, non-blinded
- Blinding: PFT results will be blinded from the treating surgical team to avoid influencing management decisions. Investigators and participants will have access to clinical data.
- Control arm: Not applicable
- Randomisation: Not applicable
- Setting: Single tertiary NHS cardiac surgery centre
- Recruitment target: 100 patients
- Follow-up: From preoperative baseline until 30 days post-CABG, with planned 1-year MACCE follow-up via digital health records

**Figure 1:**
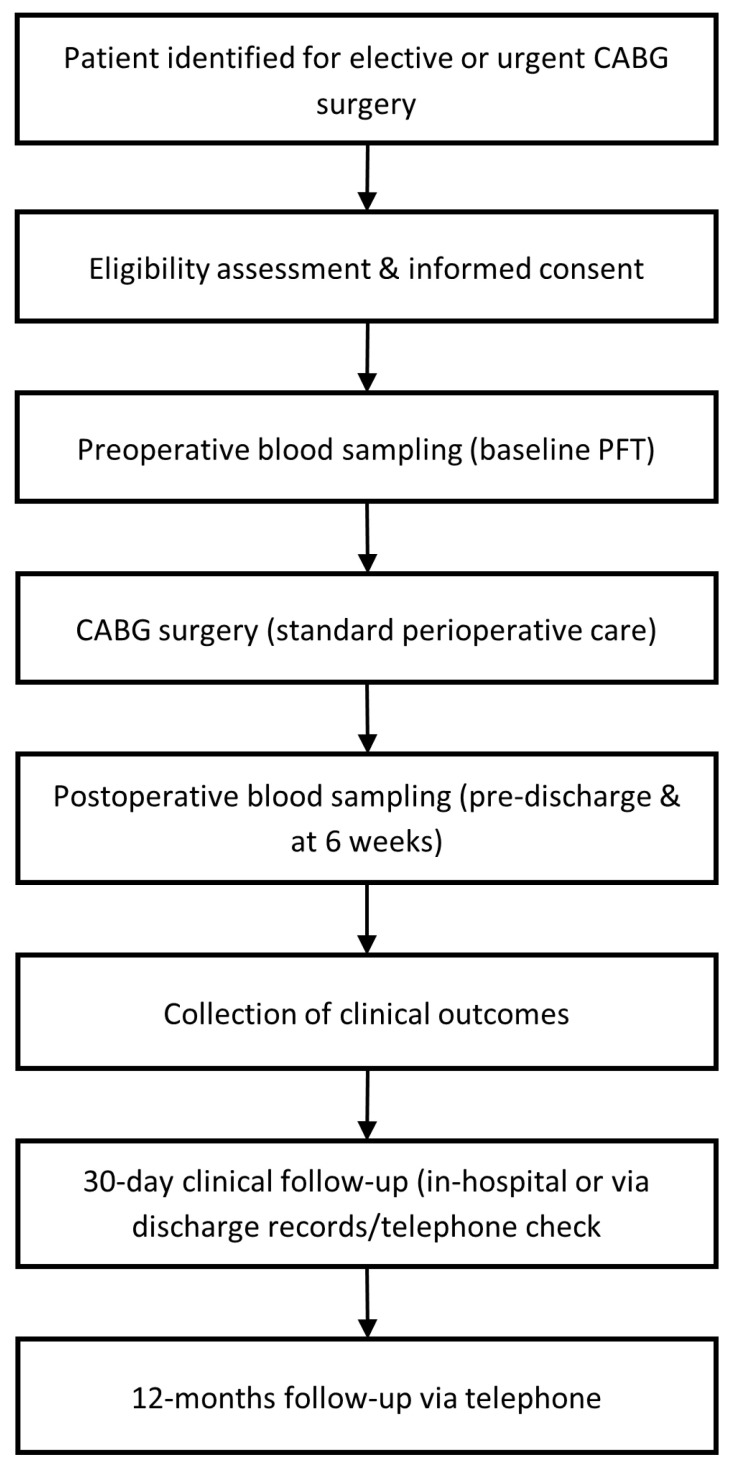
Schematic study design.

This design was chosen to capture the natural variability in platelet responsiveness and correlate these patterns with real-world surgical outcomes. Importantly, the study does not introduce any confounding influence of investigational treatments or unblinded interventions, ensuring validity in evaluating platelet function and postoperative risk stratification. Figure 1 summarizes the study design.

#### 2.1.3. Treatment and Rationale

As a non-interventional observational study, no study-specific treatment will be administered, and participants’ standard perioperative and postoperative care will remain unaltered.

All antiplatelet therapy will be prescribed at the discretion of the treating surgical teams, following institutional protocols and national guidelines. The study does not involve randomisation, dose escalation, or therapeutic modifications based on PFT results. Instead, it aims to evaluate the association between naturally occurring variability in platelet reactivity and clinical outcomes under standard care conditions.

#### 2.1.4. Standard Medications Permitted

Patients undergoing elective and urgent CABG are expected to receive the following standard antiplatelet regimens:

- Aspirin (typically 300 mg within 6 h of return from operating theatre followed by 75 mg once daily)
- Clopidogrel (typically 75 mg once daily, when prescribed)

Additionally, concomitant medications—including beta-blockers, ACE inhibitors, statins, or anticoagulants (such as warfarin or direct oral anticoagulants, DOACs)—may be prescribed as per clinical indications. These will be recorded and accounted for in the final analysis as potential confounding variables affecting platelet function and surgical outcomes.

#### 2.1.5. Significance of Study Design

This observational study provides foundational data to support the development of future clinical trials aimed at tailoring antiplatelet therapy based on individual platelet function profiles. By prospectively assessing platelet responsiveness at multiple perioperative timepoints, the study seeks to improve risk stratification, optimize therapeutic decision-making, and contribute to evolving guidelines for antiplatelet management in CABG patients.

### 2.2. Study Setting

This study will be conducted at Harefield Hospital, a leading UK cardiac surgery centre. The recruitment and clinical assessments will be performed within routine perioperative pathways, ensuring that participation does not interfere with standard patient care.

### 2.3. Participant Recruitment

Eligible patients will be identified during preoperative surgical planning, typically one to three days before their scheduled coronary artery bypass grafting. The study team will liaise with surgical and pre-assessment clinic staff to screen upcoming cases against inclusion and exclusion criteria.

Potential participants will be approached by a member of the clinical team, who will provide verbal and written information about the study. Patients will be given the Participant Information Sheet and allowed at least 24 h to consider participation unless an expedited timeline is required due to urgent surgery scheduling. Written informed consent will be obtained before any study-specific procedures, including blood sampling for PFT.

### 2.4. Eligibility Criteria

#### 2.4.1. Inclusion Criteria

- Adults aged 18 years or older
- Scheduled for elective or urgent, isolated coronary artery bypass grafting at the study centre
- Expected to receive standard antiplatelet therapy (aspirin or dual antiplatelet therapy) postoperatively as part of routine care
- Able and willing to provide written informed consent to participate in the study
- Willing to undergo perioperative blood sampling for PFT as per the study protocol
- Able to understand and comply with study procedures and follow-up requirements

#### 2.4.2. Exclusion Criteria

Participants will be excluded if they meet any of the following criteria:

- Known diagnosis of a platelet function disorder or coagulopathy (e.g., Glanzmann thrombasthenia, von Willebrand disease, myelodysplasia)
- Current use of investigational drugs or devices or participation in another interventional clinical trial within thirty days prior to enrolment
- Recent treatment within seven days with glycoprotein IIb/IIIa inhibitors (e.g., abciximab, eptifibatide), ticagrelor, prasugrel, or regular NSAIDs beyond low-dose aspirin
- Pregnancy or breastfeeding at the time of consent
- Positive pregnancy test within seven days prior to enrolment in women of childbearing potential
- Inability or unwillingness to provide written informed consent
- Severe anaemia with haemoglobin less than eight grams per decilitre or thrombocytopaenia with platelet count below one hundred times ten to the power of nine per litre at baseline
- Any condition or concurrent illness that, in the investigator’s opinion, would compromise patient safety or impair the ability to comply with study procedures

### 2.5. Study Procedures

#### 2.5.1. Platelet Function Testing

Platelet function will be assessed using TEG6s Platelet Mapping (Haemonetics), a viscoelastic assay that quantifies platelet inhibition in response to aspirin and clopidogrel therapy. Testing will be performed at three perioperative timepoints:

1. Baseline PFT will be performed on the morning before surgery.
2. Discharge at postoperative day five to seven. For patients discharged earlier than postoperative day five, testing will be undertaken prior to discharge.
3. Six-week follow-up visit

#### 2.5.2. Blood Sample Collection and Handling

- Blood samples will be obtained via peripheral venepuncture or an existing intravenous line using aseptic technique by trained personnel
- Samples will be pseudo-anonymised, labelled with a unique study ID, and stored in the operating theatres department laboratory under NHS governance
- All processing will be performed within four hours of collection in accordance with TEG6s manufacturer guidelines
- No long-term storage, banking, or genetic analysis will be performed

#### 2.5.3. Clinical Outcomes and Follow-Up

Participants will be followed from preoperative baseline through twelve months postoperatively, capturing routine clinical outcomes including:

- Major adverse cardiovascular and cerebrovascular events, including myocardial infarction, stroke, and cardiovascular death
- Postoperative bleeding, defined as Bleeding Academic Research Consortium type two or higher
- Need for surgical re-exploration for haemostasis
- Transfusion requirements, including blood product administration
- Duration of hospital stay, including intensive care unit and ward admission
- Wound healing and postoperative complications

Follow-up visits will occur at discharge between postoperative day five and seven, at six weeks postoperatively, at thirty days via telephone contact, and at twelve months through digital health records review.

### 2.6. Primary and Secondary Endpoints

#### 2.6.1. Primary Endpoint

The primary objective is to evaluate the association between perioperative platelet function test results and clinical outcomes in patients undergoing coronary artery bypass grafting.

#### 2.6.2. Secondary Endpoints

- Prevalence of aspirin and clopidogrel resistance in the study population
- Demographic and clinical predictors of antiplatelet resistance, such as age, sex, diabetes, renal function, body mass index, and surgical technique
- Temporal trends in platelet responsiveness from preoperative baseline through early and late postoperative periods
- Feasibility of integrating PFT into perioperative care pathways to support individualized antiplatelet management in future trials

### 2.7. Statistical Analysis

#### 2.7.1. Baseline Characteristics and Study Flow

Baseline characteristics will be assessed to ensure comparability across participant groups. The following variables will be evaluated:

- Demographics: Age (continuous), sex (categorical), ethnicity (categorical), BMI (continuous)
- Clinical history: Diabetes status (categorical), renal function (eGFR, continuous), hypertension (categorical), previous PCI or MI (categorical)
- Operative details: On-pump vs. off-pump surgery (categorical), number of grafts (continuous), use of antiplatelet agents (categorical)

Continuous variables will be reported as means with standard deviations or medians with interquartile ranges, depending on data distribution. Categorical variables will be presented as frequencies and percentages. Group differences will be assessed using appropriate statistical tests:

- Student’s *t*-test or Mann–Whitney U test for continuous variables
- Chi-square or Fisher’s exact test for categorical variables

#### 2.7.2. Participant Flow Reporting

Patient enrollment and study completion rates will be documented using a CONSORT-style flow diagram, adapted for observational studies. The following parameters will be reported:

- Total number of eligible patients approached
- Number who consented
- Number excluded (with reasons)
- Number completing platelet function tests at each timepoint
- Number included in endpoint analyses
- Number lost to follow-up at 6 weeks and 12 months

A study flow diagram will be included in publications, following recommendations from the STROBE statement for observational studies.

#### 2.7.3. Handling of Missing Data

Primary analyses will follow a complete case approach. If more than 10% of data is missing for a key variable, multiple imputation may be considered. All missing data patterns will be documented, and deviations will be reported in the final analysis.

#### 2.7.4. Primary Endpoint Analysis

The primary objective of this study is to evaluate the association between platelet function test results and clinical outcomes in patients undergoing elective and urgent CABG.

Summary Measures

Platelet function results will be summarized as mean ± standard deviation or median with interquartile range (IQR), depending on data distribution, for:

- AA channel (aspirin response)
- ADP channel (clopidogrel response)

Clinical outcomes, including MACCE, bleeding, re-exploration, and transfusion, will be reported as frequencies and proportions.

Method of Analysis

- Correlation analysis: Pearson’s correlation for normally distributed data, Spearman’s rank correlation for non-parametric data
- Logistic regression
- Estimates the odds of postoperative events based on platelet resistance status (resistant vs. non-resistant)
- Adjusts for potential confounders (age, diabetes, renal function, surgical factors)
- Hierarchical data structure: Generalized estimating equations (GEE) or mixed-effects models will be used to account for repeated measures of platelet function over time.

#### 2.7.5. Handling of Missing Data, Non-Compliers, and Outliers

- Complete case analysis will be the primary method.
- Multiple imputation may be used if missing data exceeds 10%.
- Outliers in platelet function tests will be assessed through visual inspection (boxplots, histograms) and verified against source data. Sensitivity analyses may exclude extreme values.

#### 2.7.6. Predefined Subgroup Analyses

Subgroup analyses will explore variations in platelet function and clinical outcomes by:

- Diabetes status (yes/no)
- Age group
- Renal function
- Clopidogrel vs. aspirin resistance
- Sex (male vs. female)

Interaction terms in regression models will be used to evaluate subgroup effects.

#### 2.7.7. Intention-to-Treat Statement

Since this is a non-randomized observational study, an intention-to-treat analysis is not applicable. Analyses will be conducted on participants with complete platelet function and outcome data.

#### 2.7.8. Secondary Endpoint Analysis

Analyses of secondary endpoints are exploratory and hypothesis-generating, as the study is not powered for statistical significance in these measures. Findings will guide future studies.

Variability in Platelet Function

- Objective: Assess inter-individual variability in platelet inhibition across timepoints.
- Analysis:
  - Continuous values (% inhibition) summarized at baseline, discharge, and 6 weeks
  - Repeated measures ANOVA or Friedman test for within-patient changes
  - Boxplots and line graphs used for visualization

Prevalence of Platelet Resistance

- Objective: Determine the proportion of patients with inadequate platelet inhibition.
- Analysis:
  - Categorical resistance status reported for each timepoint with 95% confidence intervals
  - Changes in resistance status analyzed using McNemar’s test for paired categorical data

Demographic and Clinical Predictors of Platelet Function

- Objective: Explore associations between patient characteristics and platelet responsiveness.
- Analysis:
  - Univariable and multivariable linear regression for continuous % inhibition outcomes
  - Considered variables: age, sex, BMI, renal function, diabetes, and number of grafts
  - Selection guided by clinical relevance and statistical significance (*p* < 0.1)

Impact of Platelet Resistance on Clinical Decision-Making

- Objective: Evaluate whether platelet resistance influences treatment decisions, including DAPT use and discharge timing.
- Analysis:
  - Comparisons of DAPT prescription rates between resistant and non-resistant patients using chi-square or Fisher’s exact test
  - Logistic regression to explore predictors of treatment decisions if data allows

#### 2.7.9. Handling of Multiplicity

No formal adjustments for multiple comparisons will be made, as these analyses are exploratory. All *p*-values will be interpreted cautiously, with emphasis placed on effect size and confidence intervals.

### 2.8. Study Governance and Ethical Considerations

#### 2.8.1. Committees Involved in the Study

This study is a non-randomised, observational, single-centre investigation; therefore, formal oversight by an Independent Data Monitoring Committee (IDMC) or Trial Steering Committee (TSC) is not required due to the minimal risk to participants and the non-interventional nature of the protocol. However, oversight will be provided by a Trial Management Group (TMG) to ensure adherence to Good Clinical Practice (GCP), institutional governance policies, and study protocol requirements.

The Trial Management Group (TMG) will be responsible for:

- Monitoring participant recruitment and follow-up
- Ensuring adherence to the approved study protocol
- Overseeing data quality, safety reporting, and compliance with regulatory requirements
- Addressing operational issues or protocol deviations

The TMG membership will include:

- Chief Investigator (CI)
- Study Statistician
- Study Research Nurse
- Data Manager or delegated research coordinator
- Sponsor representative (if applicable)

The TMG will meet regularly throughout the study and maintain formal records of all discussions and decisions.

#### 2.8.2. Monitoring and Auditing

The necessity for study monitoring or auditing will be determined through an internal Research Office risk assessment, following Standard Operating Procedures (SOPs). Monitoring arrangements will be proportionate to the study’s design, complexity, and risk level. If monitoring or auditing is required, visits will be arranged in advance and conducted in accordance with institutional standards, GCP guidelines, and Sponsor policies.

#### 2.8.3. Direct Access to Source Data

Study-related monitoring, audits, Research Ethics Committee (REC) review, and regulatory inspections will be permitted, ensuring direct access to source data as required. Participants will be informed of these procedures during informed consent, allowing authorised representatives (e.g., Sponsor, REC, Health Research Authority (HRA)) access to relevant sections of their medical records, in strict compliance with data protection and confidentiality regulations.

#### 2.8.4. Ethics and Regulatory Requirements

Before patient enrolment, full regulatory approval for the study will be obtained, including:

- HRA approval, incorporating REC approval
- Approval of study documents (protocol, Participant Information Sheet (PIS), Informed Consent Form (ICF), GP letter)

Recruitment will not commence until formal confirmation of capacity and capability is received from participating institutions, as outlined in HRA approval letters.

Should any substantial amendments to the protocol or study documents be necessary, they will be submitted for HRA and REC approval prior to implementation. Principal Investigators will ensure local compliance with all approvals.

In the event of premature study termination, the CI will notify both the Sponsor and REC within 15 days. A formal study closure notification will be submitted within 90 days of study completion. Additionally, a final study report summarising:

- Whether study objectives were achieved
- Main findings
- Plans for publication and dissemination

Feedback provided to study participants will be submitted to both the REC and Sponsor within 12 months of study completion.

#### 2.8.5. Finance

This study is funded by a Research Grant awarded by the Royal Brompton & Harefield Hospitals Charity, totalling £36,544.00, for a 12-month research project titled: “Evaluating Responsiveness to Antiplatelet Therapy in Patients Undergoing CABG Surgery”.

- Grant holder: Maria Comanici, Clinical Fellow, Cardiothoracic Surgery, Harefield Hospital
- Reference code: C0118: RFSG-16/24

Funding will support staff time, laboratory testing, consumables, and data management. The study has no commercial funding or sponsorship, and all financial governance will be managed in accordance with Trust and Sponsor policies, with invoicing arranged via quarterly submissions in arrears.

#### 2.8.6. Insurance and Indemnity

NHS institutions are liable for clinical negligence and other harms to patients under their duty of care. Additionally, NHS institutions employing researchers are responsible for negligent harm resulting from study design.

#### 2.8.7. Publication Policy

Data ownership will reside with the institution sponsoring the study, and study findings will be disseminated through peer-reviewed publications and conference presentations.

#### 2.8.8. Statement of Compliance

This study will be conducted in full compliance with:

- The protocol approved by the Sponsor and HRA
- Sponsor Standard Operating Procedures (SOPs)
- Good Clinical Practice (GCP)
- Relevant UK regulations, including the Human Rights Act 1998, the Data Protection Act 2018, and ethical frameworks such as the Declaration of Helsinki (2008 version)

Should any protocol deviations be required to eliminate an immediate hazard to a research subject, they will be reported to the Sponsor and REC as soon as possible.

## 3. Results

### 3.1. Participant Characteristics

Baseline demographic and clinical characteristics will be collected from enrolled participants to assess comparability between groups. Variables evaluated will include age, sex, ethnicity, body mass index, cardiovascular risk factors, preoperative medication use, and surgical approach.

### 3.2. Platelet Function and Clinical Outcomes

Platelet function test results will be analyzed at three timepoints: preoperative baseline, postoperative discharge, and six-week follow-up. The degree of platelet inhibition will be reported as percentage inhibition in response to arachidonic acid and adenosine diphosphate pathways, reflecting aspirin and clopidogrel effectiveness, respectively.

The prevalence of aspirin and clopidogrel resistance in the study population will be determined based on predefined cutoff values from the TEG6s Platelet Mapping system. Differences in platelet responsiveness between groups will be evaluated using repeated measures analysis of variance or non-parametric alternatives, depending on data distribution.

The primary analysis will assess correlations between platelet function test results and major adverse cardiovascular and cerebrovascular events, including myocardial infarction, stroke, cardiovascular death, postoperative bleeding complications, and the need for surgical re-exploration. Univariate correlations will be explored using Pearson or Spearman correlation coefficients, while logistic regression models will estimate the odds ratios for adverse outcomes associated with platelet resistance.

### 3.3. Secondary Analyses

Secondary analyses will investigate potential predictors of platelet function variability, including demographic and clinical factors such as diabetes status, renal function, smoking history, and surgical approach. Multivariable regression models will be employed to adjust for key covariates and assess independent associations with platelet responsiveness.

Additionally, the feasibility of integrating PFT into routine perioperative workflows will be examined. The study will evaluate whether PFT results influence clinical decision-making, including modifications in antiplatelet therapy or postoperative management.

## 4. Discussion

This study aims to address the critical challenge of antiplatelet resistance in patients undergoing coronary artery bypass grafting (CABG). Despite the widespread use of dual antiplatelet therapy, a significant proportion of patients exhibit laboratory-confirmed resistance to aspirin and/or clopidogrel. This resistance, whether transient due to perioperative platelet turnover or persistent due to genetic, metabolic, or inflammatory mechanisms, has been associated with adverse cardiovascular outcomes such as graft occlusion, myocardial infarction, and increased mortality risk [5,6].

Current guidelines do not recommend routine PFT for CABG patients, primarily due to a lack of high-quality prospective data correlating platelet reactivity profiles with postoperative outcomes [9,10]. As a result, postoperative antiplatelet management remains largely empirical, contributing to variability in prescribing practices and uncertainty regarding the role of PFT [8]. The present study seeks to address this gap by prospectively evaluating perioperative platelet reactivity and correlating these findings with clinical outcomes to determine whether PFT can serve as a reliable risk stratification tool for CABG patients.

## 5. Potential Implications

By systematically assessing platelet function in the perioperative period, this study may provide critical insights into the prognostic relevance of antiplatelet resistance. Prior observational research suggests that pre- and postoperative platelet reactivity profiles may serve as early indicators of risk, enabling clinicians to identify patients at heightened risk for ischemic complications [5,11]. Additionally, studies have demonstrated that conventional aspirin doses (100 mg daily) may be insufficient in suppressing thromboxane synthesis in the early postoperative period due to accelerated platelet turnover [11,12]. The potential for transient resistance to resolve spontaneously, as reported in previous investigations, highlights the dynamic nature of platelet responsiveness and underscores the importance of individualized antiplatelet strategies [12].

Beyond individual risk stratification, the findings from this study may inform broader clinical practice by guiding the development of personalized antiplatelet regimens tailored to patients’ pharmacodynamic profiles. Previous studies, such as the BOCLA-Plan and tailored dual antiplatelet strategies using CYP2C19 genotyping, have demonstrated that dose adjustments or alternative antiplatelet agents can effectively overcome resistance [13,14]. If the present study validates the predictive utility of PFT, it could provide the foundation for future randomized trials evaluating personalized antiplatelet approaches, ultimately improving outcomes in CABG patients.

## 6. Study Limitations

As an observational study, this research does not involve direct therapeutic interventions by the study team. However, PFT results will be conveyed to the clinical team caring for the patient, who may modify treatment based on their clinical judgment. The study itself will generate valuable data regarding platelet function trends and potential predictors of poor outcomes, contributing to a better understanding of antiplatelet resistance without directly altering patient management within the study framework.

This study does not include immediate postoperative PFT upon arrival to the intensive care unit. While such measurements may offer mechanistic insights, discharge sampling was selected to balance clinical feasibility, patient safety, and assessment of stabilized platelet function following early recovery and antiplatelet therapy initiation.

Additionally, as with all studies incorporating PFT, variability in assay methodology and interpretation remains a challenge, given the lack of universal standardization across testing platforms [6]. Furthermore, the study is designed to evaluate platelet function within a defined perioperative timeframe, yet long-term outcomes beyond the study period may also be influenced by additional variables such as medication adherence, comorbid conditions, and secondary prevention strategies.

While venesection poses minimal risk, the additional blood sampling may introduce slight inconvenience for participants, although efforts have been made to integrate sample collection within routine clinical care.

## 7. Future Directions

Inter-individual variability in platelet responsiveness is influenced not only by clinical and perioperative factors but also by genetic determinants. Single nucleotide polymorphisms affecting platelet receptor pathways and antiplatelet drug metabolism—most notably variants in genes such as CYP2C19 involved in clopidogrel activation—have been shown to contribute to reduced antiplatelet efficacy and adverse cardiovascular outcomes. Integration of pharmacogenomic testing with platelet function assessment may therefore offer a more comprehensive approach to individualized antiplatelet therapy. Although genetic testing was beyond the scope of the present study due to its observational design and regulatory considerations, future studies incorporating combined platelet function and pharmacogenomic profiling may further refine risk stratification and therapeutic decision-making in CABG patients.

Should the study demonstrate a robust correlation between perioperative platelet function and postoperative outcomes, the findings may pave the way for future large-scale trials assessing the clinical utility of PFT-guided antiplatelet strategies. Specifically, randomized investigations evaluating tailored therapy based on individual platelet reactivity profiles could help bridge the existing gap between mechanistic understanding and therapeutic decision-making. Additionally, long-term follow-up studies examining the durability of platelet function trends beyond the immediate postoperative phase may further elucidate the trajectory of antiplatelet resistance and refine patient management strategies.

Taken together, this study is expected to contribute meaningful insights into the evolving field of CABG pharmacotherapy. By systematically analyzing platelet function patterns and correlating them with postoperative outcomes, this investigation holds the potential to advance precision medicine in surgical myocardial revascularisation, ultimately improving patient safety and optimizing antiplatelet management in CABG populations.

## Data Availability

All data collected for the study, including study protocol, individual participant data, and a data dictionary defining each field in the set, will be made available to others; deidentified participant data will be available after the publication of the final manuscript from the corresponding author (maria.comanici1@nhs.net); after approval of a proposal, with a signed data access agreement, with restrictions on sharing this data with a third party without written approval of corresponding author.
